# Synthesis of Nitrogen‐Doped KMn_8_O_16_ with Oxygen Vacancy for Stable Zinc‐Ion Batteries

**DOI:** 10.1002/advs.202106067

**Published:** 2022-02-10

**Authors:** Guodong Cui, Yinxiang Zeng, Jinfang Wu, Yan Guo, Xiaojun Gu, Xiong Wen (David) Lou

**Affiliations:** ^1^ School of Chemistry and Chemical Engineering Inner Mongolia University Hohhot 010021 China; ^2^ School of Chemical and Biomedical Engineering Nanyang Technological University 62 Nanyang Drive Singapore 637459 Singapore

**Keywords:** aqueous zinc‐ion batteries, KMn_8_O_16_, N‐doping, oxygen vacancy

## Abstract

The development of MnO_2_ as a cathode for aqueous zinc‐ion batteries (AZIBs) is severely limited by the low intrinsic electrical conductivity and unstable crystal structure. Herein, a multifunctional modification strategy is proposed to construct N‐doped KMn_8_O_16_ with abundant oxygen vacancy and large specific surface area (named as N‐KMO) through a facile one‐step hydrothermal approach. The synergetic effects of N‐doping, oxygen vacancy, and porous structure in N‐KMO can effectively suppress the dissolution of manganese ions, and promote ion diffusion and electron conduction. As a result, the N‐KMO cathode exhibits dramatically improved stability and reaction kinetics, superior to the pristine MnO_2_ and MnO_2_ with only oxygen vacancy. Remarkably, the N‐KMO cathode delivers a high reversible capacity of 262 mAh g^−1^ after 2500 cycles at 1 A g^−1^ with a capacity retention of 91%. Simultaneously, the highest specific capacity can reach 298 mAh g^−1^ at 0.1 A g^−1^. Theoretical calculations reveal that the oxygen vacancy and N‐doping can improve the electrical conductivity of MnO_2_ and thus account for the outstanding rate performance. Moreover, ex situ characterizations indicate that the energy storage mechanism of the N‐KMO cathode is mainly a H^+^ and Zn^2+^ co‐insertion/extraction process.

## Graphical abstract

A multifunctional modification strategy is developed to construct N‐doped KMn_8_O_16_ with abundant oxygen vacancy and large specific surface area (N‐KMO). The synergistic effects of distinctive structures and compositions can effectively facilitate the transport of electron/ions and suppress the dissolution of manganese ions. As a result, the N‐KMO cathode exhibits dramatically improved cycling stability and rate performance for aqueous zinc‐ion batteries.

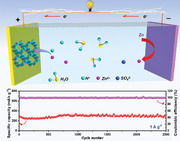

## 1 Introduction

The growing environmental concerns and energy consumption are driving an ever‐increasing pursuit for advanced energy storage system with high energy density, environmental friendliness, and high safety.^[^ ^1^ ^]^ Although lithium‐ion batteries (LIBs) dominate the battery market due to their light weight, high energy density, and long cycle life, the application of LIBs as large‐scale energy storage systems has been plagued by the safety issues and environmental problems associated with flammable organic electrolytes.^[^ ^2^, ^3^ ^]^ In recent years, aqueous rechargeable batteries, which feature high safety, eco‐friendliness, and high ion conductivity of water‐based electrolyte have been considered as promising alternatives to overcome these dilemma.^[^ ^4^ ^]^ Particularly, aqueous zinc‐ion batteries (AZIBs) using Zn anode has shown significant promise for grid‐scale energy storage owing to the high specific capacity (820 mAh g^−1^), low redox potential, rich abundance, and low cost of metallic Zn.^[^ ^5^, ^6^, ^7^, ^8^ ^]^ Nevertheless, the further development of next‐generation AZIBs is mainly hindered by cathode materials. A significant challenge remains to construct highly reversible cathode materials with good electrochemical properties.^[^ ^9^, ^10^, ^11^, ^12^ ^]^

The current cathode materials for AZIBs mainly include manganese‐based materials,^[^ ^13^, ^14^, ^15^, ^16^ ^]^ vanadium‐based materials,^[^ ^17^, ^18^, ^19^, ^20^, ^21^ ^]^ Prussian blue and its analogs,^[^ ^22^, ^23^ ^]^ and organic compounds.^[^ ^24^, ^25^, ^26^ ^]^ Among them, MnO_2_ characterized by high theoretical capacity (308 mAh g^−1^, contributed capacity of single electron transfer), cost‐effectiveness, high natural abundance, and environmental friendliness has attracted extensive scientific attention.^[^ ^27^ ^]^ Moreover, because of the favorable 2 × 2 tunnels with size of 4.6 Å, *α*‐MnO_2_ is considered as a compelling cathode material for AZIBs.^[^ ^28^ ^]^ However, most reported *α*‐MnO_2_ suffer from poor electric conductivity (≈10^−5^ to 10^−6^ S cm^−1^), structural damage, and dissolution caused by unstable crystal structure and the Jahn–Teller distortion of Mn^3+^ ions, eventually resulting in inferior rate performance and rapid capacity attenuation during cycling.^[^ ^27^, ^29^, ^30^, ^31^ ^]^

To address these issues, strategies including compositing conductive materials (e.g., carbon materials and conductive polymers),^[^ ^32^ ^]^ structural design, and defect engineering (e.g., oxygen vacancy, pre‐intercalation of metal cations, or non‐metal ions doping) have been extensively studied with some promising progress.^[^ ^33^, ^34^, ^35^, ^36^ ^]^ It is well known that oxygen vacancy can not only increase the electrical conductivity of metal oxides, but also regulate the electrochemical activity and promote ion diffusion.^[^ ^34^ ^]^ The pre‐intercalation of K^+^ can improve the structural stability of MnO_2_.^[^ ^33^, ^37^, ^38^ ^]^ Furthermore, heteroatom doping can effectively adjust the intrinsic characteristics of materials such as electron/ion transfer, adsorption property, reaction activity, and structural stability.^[^ ^31^, ^39^, ^40^, ^41^, ^42^ ^]^ Some inspiring studies have employed these strategies to boost the zinc ion storage performance of MnO_2_.^[^ ^43^, ^44^, ^45^, ^46^, ^47^, ^48^, ^49^, ^50^ ^]^ However, most researches involve complex multi‐step synthesis methods and more explorations are needed to further optimize the electrochemical performance. Therefore, it is highly desirable to introduce oxygen vacancy and heteroatom doping into MnO_2_ simultaneously via a facile and efficient approach.

In this work, N‐doped KMn_8_O_16_ with abundant oxygen vacancy, large specific surface area, and pore volume (named as N‐KMO) is rationally designed and synthesized by an efficient one‐step hydrothermal method. The introduced oxygen vacancy, pre‐intercalated K^+^, N‐doping, large specific surface area, and pore volume can play a vital role in improving the electrical conductivity of N‐KMO and facilitating the ion adsorption and diffusion, contributing to enhanced rate capability. Moreover, the N‐doping into the bulk phase of N‐KMO can effectively improve the structural stability by inhibiting the Jahn–Teller distortion of Mn^3+^ derived from the discharge product. Benefiting from these synergic effects, the N‐KMO cathode exhibits long cycle life and superior rate performance. Meanwhile, the energy storage mechanism is proved to be a H^+^ and Zn^2+^ co‐insertion/extraction process by various ex situ characterizations.

## 2 Results and Discussion

### 2.1 Physical Characterizations

**Figure 1:**
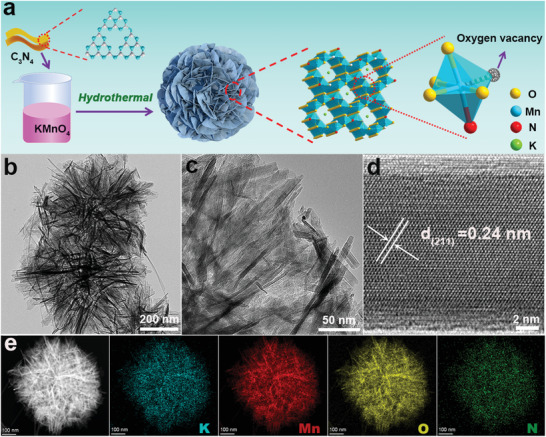
a) The synthesis schematic diagram, b,c) TEM images, d) HRTEM image, e) HAADF‐STEM and the corresponding elemental mapping images of N‐KMO.

As shown in the synthesis schematic diagram (**Figure** **1**a), N‐KMO was synthesized through an efficient one‐step hydrothermal redox reaction between KMnO_4_ and C_3_N_4_. Two‐dimensional C_3_N_4_ nanosheets (Figure S1, Supporting Information) were used here as both reducing agent and nitrogen source . C_3_N_4_ may be dissociated under hydrothermal reaction at 180 °C, and fully react with KMnO_4_ through oxidation–reduction reaction to generate N‐KMO. For comparison, *α*‐MnO_2_ with oxygen vacancy (named as O_v_‐MnO_2‐_ *_x_*, Figure S2a, Supporting Information) and pristine MnO_2_ (Figure S2b, Supporting Information) were also prepared. The scanning electron microscopy (SEM) and transmission electron microscopy (TEM) images indicate that N‐KMO shows a nanoflowers morphology assembled by interconnected nanosheets (Figure 1b,c; Figure S3, Supporting Information). In the high‐resolution TEM (HRTEM) image (Figure 1d), the lattice spacing of 0.24 nm can be clearly observed, which is assigned to the (211) crystal plane of KMn_8_O_16_ (PDF#29‐1020). The TEM image and the corresponding selected area electron diffraction (SAED) pattern (Figure S4, Supporting Information) exhibit typical polycrystalline diffraction rings, which can be indexed to the KMn_8_O_16_ phase. The high‐angle annular dark‐field scanning TEM image (HAADF‐STEM) and the corresponding elemental mapping images of the N‐KMO (Figure 1e) reveal that the N, O, K, and Mn elements are evenly distributed in the nanoflowers, which demonstrates the existence of N element.

**Figure 2:**
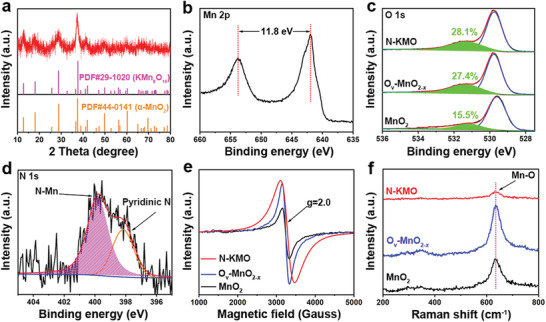
a) XRD pattern of N‐KMO. High‐resolution XPS spectra of b) Mn 2p, c) O 1s, and d) N 1s for N‐KMO. e) EPR curves and f) Raman spectra of N‐KMO, O_v_‐MnO_2‐_ *_x_*, and MnO_2_.

The X‐ray diffraction (XRD) pattern of N‐KMO indicates that the characteristic diffraction peaks of C_3_N_4_, located at 13.6° and 27.2°, completely disappear after the hydrothermal process (**Figure** **2**a; Figure S5, Supporting Information). In addition, the XRD patterns of N‐KMO, O_v_‐MnO_2‐_ *_x_*, and MnO_2_ have similar crystallographic structure of tetragonal *α*‐MnO_2_ phase (PDF#44‐0141). Simultaneously, the XRD diffraction peaks of N‐KMO correspond well to KMn_8_O_16_ (PDF#29‐1020). The inductively coupled plasma optical emission spectroscopy (ICP‐OES) shows that the molar ratio of Mn:K is about 8:1 in N‐KMO, which demonstrates that K^+^ is successfully pre‐intercalated into the tunnels of N‐KMO to improve structural stability.^[^ ^33^, ^37^, ^38^ ^]^ The diffraction peaks of N‐KMO are significantly broadened and weakened compared to that of MnO_2_, indicating the lower crystallinity of N‐KMO, which may be caused by the N‐doping. The valence state and chemical composition of the N‐KMO were investigated by X‐ray photoelectron spectroscopy (XPS). The XPS survey spectrum (Figure S6, Supporting Information) suggests the existence of Mn, O, N, and K elements in the N‐KMO sample, consistent with elemental mapping result. In the high‐resolution Mn 2p spectrum (Figure 2b), the peaks of Mn 2p_3/2_ and Mn 2p_1/2_ with a spin‐energy separation of 11.8 eV for typical MnO_2_ appear at 641.9 eV and 653.7 eV, respectively.^[^ ^35^ ^]^ At the same time, the spin‐energy separation of 4.8 eV appears in the high‐resolution spectrum of Mn 3s (Figure S7, Supporting Information), indicating that the valence state of N‐KMO is mainly Mn^4+^.^[^ ^51^ ^]^ The high‐resolution O 1s spectra can be deconvoluted into two peaks at 531.2 and 529.7 eV attributed to oxygen vacancy and Mn—O—Mn bond, respectively (Figure 2c).^[^ ^36^ ^]^ Obviously, the ratio of oxygen vacancy peak (calculated from the integrated area in the fitted spectra) for N‐KMO and O_v_‐MnO_2‐_ *_x_* is higher than that of MnO_2_, revealing the enhanced oxygen vacancy in N‐KMO and O_v_‐MnO_2‐_ *_x_*. Two characteristic peaks appear at 399.7 and 398.1 eV in the N 1s spectrum (Figure 2d), corresponding to the N‐Mn bond and pyridine N, respectively.^[^ ^52^ ^]^ The formation of N‐Mn bond proves that N is successfully doped into the bulk phase of N‐KMO, and the pyridine N plays an important role in improving the conductivity. The mass ratio of N is around 2.63% based on the elemental analysis test. The K 2p XPS spectrum of N‐KMO (Figure S8, Supporting Information) again proves the existence of K element.

The electron paramagnetic resonance (EPR) spectra (Figure 2e) exhibit that N‐KMO and O_v_‐MnO_2‐_ *_x_* display much stronger signal at *g* = 2.0 than that of MnO_2_. This indicates the presence of abundant oxygen vacancy in N‐KMO and O_v_‐MnO_2‐_ *_x_*,^[^ ^35^ ^]^ which is consistent with the results of XPS. The existence of oxygen vacancies in N‐KMO is also confirmed by HRTEM images (Figure S9, Supporting Information). Moreover, the Raman spectra (Figure 2f) show that the peak located at 635 cm^−1^ is assigned to the vibration of Mn‐O bond for N‐KMO, O_v_‐MnO_2‐_ *_x_*, and MnO_2_.^[^ ^53^, ^54^ ^]^ To evaluate the surface area and volume of pores, nitrogen absorption and desorption tests were performed. As shown in Figures S10–S12 (Supporting Information), the N‐KMO possesses higher Brunauer–Emmett–Teller (BET) specific surface area (157.45 m^2^ g^−1^) and volume of pores (0.92 cm^3^ g^−1^) than O_v_‐MnO_2‐*x*_ (26.43 m^2^ g^−1^, 0.05 cm^3^ g^−1^) and MnO_2_ (25.94 m^2^ g^−1^, 0.05 cm^3^ g^−1^), which could increase the active sites and promote the ions diffusion, thus boosting the energy storage performance.

**Figure 3:**
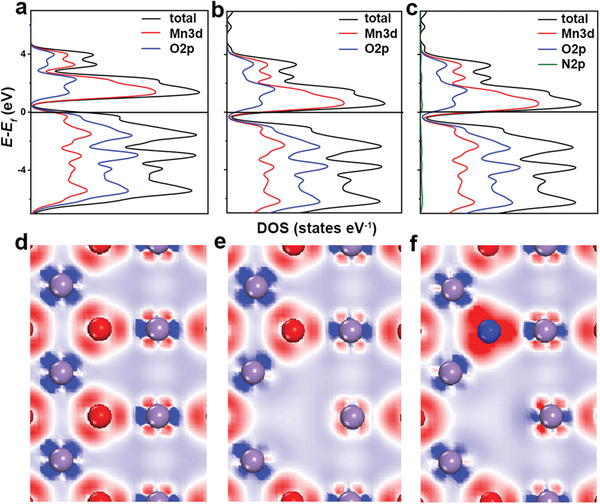
The total DOS and PDOS of a) MnO_2_, b) O_v_‐MnO_2‐_ *_x_*, and c) N‐MnO_2‐_ *_x_*. The electron density difference slices of d) MnO_2_, e) O_v_‐MnO_2‐_ *_x_*, and f) N‐MnO_2‐_ *_x_*. The red and blue areas represent the accumulation and depletion of electrons, respectively. And the purple, red, and blue balls represent the Mn, O, and N, respectively.

Density functional theory (DFT) calculations were conducted using MnO_2_, O_v_‐MnO_2‐_ *_x_*, and N­‐doped MnO_2_ with oxygen vacancy (N‐MnO_2‐_ *_x_*) as the models to verify the effect of oxygen vacancy and N‐­doping (Figure S13, Supporting Information). The calculated total density of states (DOS) and partial density of states (PDOS) of MnO_2_, O_v_‐MnO_2‐_ *_x_*, and N‐MnO_2‐_ *_x_* are illustrated in **Figure** **3**a–c. Compared with MnO_2_, the DOS of O_v_‐MnO_2‐_ *_x_* moves to low energy direction with the Fermi level entering the conduction band, leading to easier jump of electrons from the valence band to the conduction band. This implies that the introduction of oxygen vacancy could improve the electronic conductivity of MnO_2_. After doping N atom, the DOS of N‐MnO_2‐_ *_x_* further moves to lower energy, demonstrating much better electronic conductivity. Moreover, the charge density differences were presented to visualize the effect of oxygen vacancy and N‐doping on the electronic rearrangement (Figure 3d–f). Compared with MnO_2_, the Mn atoms adjacent to oxygen vacancy and doped N atom show obvious electron accumulation, which suggests strong interaction between interconnected Mn, O, and N atoms.

### 2.2 Electrochemical Performance

**Figure 4:**
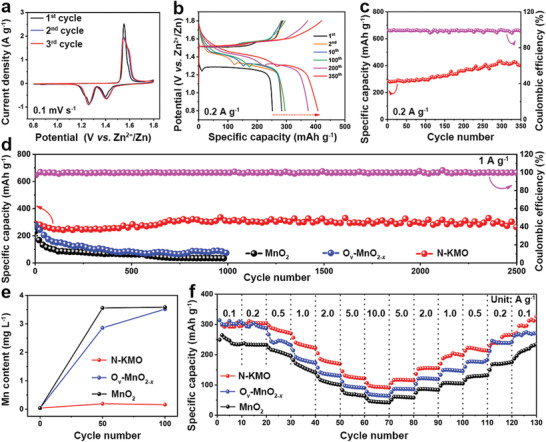
Electrochemical performance of AZIBs based on the N‐KMO, O_v_‐MnO_2‐_ *_x_*, and MnO_2_ cathodes. a) CV curves at a scan rate of 0.1 mV s^−1^, b) GCD curves at a current density of 0.2 A g^−1^, and c) the corresponding cycling performance and Coulombic efficiency of N‐KMO. d) Cycling performance at a current density of 1 A g^−1^. e) Mass content of dissolved Mn element in a 2 m ZnSO_4_ aqueous electrolyte during cycling. f) Rate performance.

The electrochemical performance of these samples was evaluated as cathode materials for AZIBs. **Figure** **4**a shows the cyclic voltammetry (CV) curves of the N‐KMO cathode at a scan rate of 0.1 mV s^−1^. Two couples of redox peaks can be observed at 1.59/1.40 V and 1.55/1.27 V, respectively. The well‐maintained profiles in the subsequent cycles suggest good electrochemical stability. Figure 4b presents the galvanostatic charge/discharge (GCD) curves of the N‐KMO cathode tested at a current density of 0.2 A g^−1^. There are two obvious charging (1.6 V and 1.5 V) and discharging (1.4 V and 1.3 V) platforms, in line with the CV results. The two discharging plateaus might correspond to the insertion of H^+^ and Zn^2+^, respectively.^[^ ^32^ ^]^ Impressively, the specific capacity of N‐KMO increases gradually upon cycling. This may be due to the fact that the N‐KMO cathode is activated at a low current density and the Mn^2+^ in the electrolyte is deposited on the N‐KMO cathode.^[^ ^43^ ^]^ As shown in Figure S14 (Supporting Information), the specific capacity and capacity contribution ratio of the first discharge platform gradually increase upon cycles. This indicates that the increase in specific capacity mainly comes from the contribution of the first discharge platform. The corresponding cycling stability of the N‐KMO cathode is shown in Figure 4c. The discharge capacity increased from 285 to 407 mAh g^−1^ after 350 cycles, accompanied with an average Coulombic efficiency of 98%, indicating a remarkable cycling durability. Moreover, the cycling stability of the N‐KMO electrode was further tested at a current density of 1 A g^−1^ (Figure 4d). The N‐KMO cathode delivers an initial discharge capacity of 288 mAh g^−1^ and a high reversible capacity of 262 mAh g^−1^ after 2500 cycles with a capacity retention of 91%. Meanwhile, the corresponding Coulombic efficiency is always approaching 100%. For comparison, the specific capacities of both O_v_‐MnO_2‐_ *_x_* and MnO_2_ cathodes have different degrees of deterioration during cycling (Figure 4d), which is mainly caused by the dissolution of manganese ions, as confirmed by the results of ICP‐OES (Figure 4e). The superior cycling stability of N‐KMO over O_v_‐MnO_2‐_ *_x_* and MnO_2_ could be attributed to the N‐doping, which effectively inhibits the dissolution of manganese ions caused by the Jahn–Teller distortion of Mn^3+^ ions from the discharged products.^[^ ^29^, ^30^ ^]^

The rate performance of the N‐KMO, O_v_‐MnO_2‐_ *_x_* and MnO_2_ cathodes is shown in Figure 4f. At a low current density of 0.1 A g^−1^, the initial discharge specific capacity of the N‐KMO cathode is 298 mAh g^−1^. As current density increases to 10 A g^−1^, a considerable average specific capacity of 106 mAh g^−1^ is still retained, revealing the superior rate capability. Simultaneously, the specific capacity can reach 327 mAh g^−1^ when the current density is restored to 0.1 A g^−1^. Remarkably, the N‐KMO cathode shows better rate performance than the O_v_‐MnO_2‐_ *_x_* and MnO_2_ cathodes, as well as most reported Mn‐based cathodes for AZIBs (as shown in Table S1, Supporting Information). This may be mainly attributed to that the oxygen vacancy, N‐doping, and large specific surface area and pore volume facilitate the ions diffusion and electron conduction, thus boosting the reaction kinetics of N‐KMO. Additionally, the cyclic and rate performances of the N‐KMO cathode with high mass loading (around 3.5 mg cm^−2^) are also evaluated (Figure S15, Supporting Information), which are comparable to those of N‐KMO cathode with low mass loading (around 1 mg cm^−2^) and other reported film electrodes.^[^ ^46^, ^55^ ^]^ Furthermore, N‐KMO also exhibits higher energy density and power density (297.0 Wh kg^−1^ at 503.0 W kg^−1^; 106.0 Wh kg^−1^ at 10.6 kW kg^−1^) than O_v_‐MnO_2‐_ *_x_*, MnO_2_ and other reported materials (Figure S16, Supporting Information, *β*‐MnO_2_;^[^ ^56^ ^]^ CuHCF;^[^ ^57^ ^]^ ZnHCF;^[^ ^58^ ^]^ *δ*‐MnO_2_;^[^ ^59^ ^]^ Zn_0.25_V_2_O_5_^[^ ^60^ ^]^).

**Figure 5:**
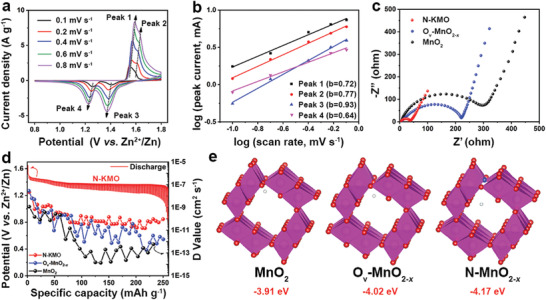
Electrochemical kinetics of the N‐KMO cathode. a) CV curves at different scan rates. b) Determination of the *b* values using the relationship between peak current and scan rate. c) Nyquist plots, and d) the discharge GITT of N‐KMO and the diffusion coefficient of N‐KMO, O_v_‐MnO_2‐_ *_x_*, and MnO_2_. e) H^+^ intercalation energy in N‐MnO_2‐_ *_x_*, O_v_‐MnO_2‐_ *_x_*, and MnO_2_ (The white ball represents the H^+^).

To reveal the charge storage kinetics, CV was tested for the N‐KMO and O_v_‐MnO_2‐_ *_x_* cathodes at different scan rates (**Figure** **5**a; Figure S17a, Supporting Information). The relationship between peak current (*i*) and scan rate (*v*) follows *i* = a*v* ^b^, where the values of *a* and *b* are two empirical constants. When the *b* values are 0.5 and 1, suggesting that the reaction is mainly controlled by the diffusion control process and the surface capacitance effect, respectively.^[^ ^36^ ^]^ As shown in Figure 5b, the *b* values of peaks 1, 2, 3, and 4 for N‐KMO are 0.72, 0.77, 0.93, and 0.64, respectively. This indicates that the electrochemical reaction of the N‐KMO cathode is controlled by both diffusion and capacitance. Meanwhile, all the *b* values for N‐KMO are higher than that of O_v_‐MnO_2‐_ *_x_* (Figure S17b, Supporting Information), implying the better reaction kinetics for the N‐KMO cathode.

The electrochemical impedance spectroscopy (EIS) and galvanostatic intermittent titration technique (GITT) tests were further performed to evaluate the electrochemical kinetics. As expected, the charge transfer resistance of the N‐KMO electrode is much lower than that of the O_v_‐MnO_2‐_ *_x_* and MnO_2_ electrodes (Figure 5c), demonstrating that the N‐doping and oxygen vacancy can facilitate the electron transport. In addition, the electrical conductivity of N‐KMO powder (8 × 10^−4^ S cm^−1^) is obviously higher than that of the reported *α*‐MnO_2_ (≈10^−5^ to 10^−6^ S cm^−1^).^[^ ^31^ ^]^ Moreover, the diffusion coefficient (*D* value) of the N‐KMO electrode (10^−11^ to 10^−7^ cm^2^ s^−1^) is higher than that of the O_v_‐MnO_2‐_ *_x_* and MnO_2_ electrodes (Figure 5d), suggesting the faster ions diffusion of N‐KMO. Figure 5e compares the insertion energies of H^+^ in MnO_2_, O_v_‐MnO_2‐_ *_x_* and N‐MnO_2‐_ *_x_*. The insertion energy is in the order of MnO_2_ > O_v_‐MnO_2‐_ *_x_* > N‐MnO_2‐_ *_x_*, indicating that the co‐introduction of oxygen vacancy and N dopant could facilitate the insertion of H^+^. These above results indicate the multifunctional modifications and optimal structure (that is, N‐doping, oxygen vacancy, pre‐intercalation of K^+^, suitable porous structure, and large specific surface area) endow the N‐KMO electrode with enhanced electron and ion transfer ability, as well as improved structural stability, thereby achieving outstanding cycling and rate performance.

### 2.3 Energy Storage Mechanism

**Figure 6:**
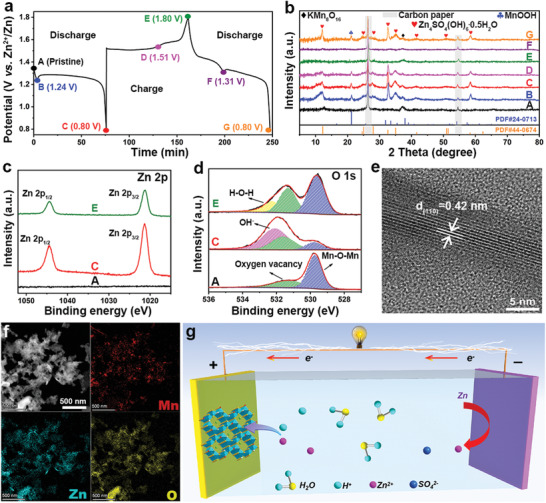
Energy storage mechanism of the N‐KMO cathode. a) The GCD profiles of the first and second cycles at 0.2 A g^−1^ and b) the corresponding ex situ XRD patterns. The ex situ high‐resolution XPS spectra of c) Zn 2p and d) O 1s. e) The ex situ HRTEM image of the N‐KMO electrode after the first full discharge. f) The ex situ HAADF‐STEM and the corresponding elemental mapping images at fully discharged state. g) Schematic for the possible working principle of Zn//N‐KMO AZIBs.

The exploration of energy storage mechanism of manganese‐based cathode for AZIBs is of great significance but still a huge challenge. Generally, the energy storage mechanisms of manganese‐based cathodes mainly include the following:^[^ ^9^ ^]^ traditional Zn^2+^ insertion/extraction,^[^ ^61^ ^]^ H^+^ and Zn^2+^ co‐insertion,^[^ ^62^ ^]^ chemical conversion reaction,^[^ ^63^ ^]^ combined intercalation and conversion reaction,^[^ ^64^ ^]^ and dissolution‐deposition mechanism.^[^ ^65^ ^]^ To explore the energy storage mechanism of the N‐KMO cathode, ex situ XRD, XPS, HRTEM, and elemental mappings were performed during the charge–discharge processes. The GCD profiles of the first and second cycles at 0.2 A g^−1^ and the corresponding ex situ XRD patterns are shown in **Figure** **6**a,b. In the pristine XRD pattern, there are three obvious peaks corresponding to the carbon paper (26.3° and 54.5°) and KMn_8_O_16_ (37.2°). It can be clearly observed that the diffraction peaks of Zn_4_SO_4_(OH)_6_^.^0.5H_2_O (PDF#44‐0674) and MnOOH phase (PDF#24‐0713) appear after discharge to 1.24 V (point B) and disappear after full charging (point E), which proves the reversible formation/decomposion of MnOOH and Zn_4_SO_4_(OH)_6_^.^0.5H_2_O products in the first voltage platform. The above electrochemical reaction involves H^+^ insertion, which corresponds to the first discharge platform.^[^ ^32^, ^62^ ^]^ As shown in the Zn 2p high‐resolution XPS spectra (Figure 6c), no Zn element is detected in the pristine N‐KMO, but two obvious Zn 2p peaks appear at a fully discharged state (point C) and weaken after full charging (point E), further confirming the formation/decomposion of Zn_4_SO_4_(OH)_6_·0.5H_2_O and the insertion/extraction of Zn^2+^ during the discharge–charge processes. The existence of Zn signal in fully charged state (point E) may be caused by the surface adsorbed or lattice trapped Zn^2+^.^[^ ^66^ ^]^ In the O 1s high‐resolution XPS spectra (Figure 6d), an obvious OH^−^ peak (at 531.9 eV)^[^ ^67^ ^]^ appears in a fully discharged state, which may be derived from Zn_4_SO_4_(OH)_6_·0.5H_2_O and MnOOH, and it recovers to the original state after being fully charged.

Furthermore, the ex situ HRTEM and elemental mappings were performed to further study the reaction mechanism of N‐KMO. As characterized in Figure 6e, a lattice fringe of 0.42 nm is observed for N‐KMO at the first discharged state, which can correspond to (110) plane of MnOOH, in accordance with the XRD results. This result further proves the H^+^ insertion during the discharge process. According to the HAADF‐STEM and the corresponding elemental mapping images of N‐KMO at the first discharged state (Figure 6f), obvious Zn signals appear together with Mn and O, which further proves the Zn^2+^ insertion. Based on the above results, the energy storage mechanism of N‐KMO is mainly a H^+^ and Zn^2+^ co‐insertion/extraction process during the discharge‐charge processes (Figure 6g).

## 3 Conclusions

In summary, we have designed and synthesized multifunctional modified N‐KMO through an efficient hydrothermal strategy. Benefiting from the synergetic effects of the enhanced electron and ion transfer ability, as well as the structural stability, the N‐KMO is employed as a highly stable and high‐rate cathode for AZIBs, and exhibits outstanding cycling and rate performance. Specifically, the N‐KMO cathode shows a high capacity of 262 mAh g^−1^ with a capacity retention of 91% after 2500 cycles at 1 A g^−1^. Moreover, a reversible capacity of 106 mAh g^−1^ at a high current density of 10 A g^−1^ and a highest power density of 10.6 kW kg^−1^ are both achieved by the N‐KMO cathode. In addition, the ex situ characterizations reveal that the energy storage mechanism of the N‐KMO cathode is mainly a H^+^ and Zn^2+^ co‐insertion/extraction process.

## Conflict of Interest

The authors declare no conflict of interest.

## Supporting information

Supporting Information

## Data Availability

The data that support the findings of this study are available from the corresponding author upon reasonable request.

## References

[1] J. B. Goodenough , Energy Environ. Sci. 2014, 7, 14.

[2] J. B. Goodenough , K. S. Park , J. Am. Chem. Soc. 2013, 135, 1167. 23294028 10.1021/ja3091438

[3] Y. Huang , Y. Fang , X. F. Lu , D. Luan , X. W. Lou , Angew. Chem. Int. Ed. 2020, 59, 19914. 10.1002/anie.202008987 32697016

[4] J. Song , K. Xu , N. Liu , D. Reed , X. Li , Mater. Today 2021, 45, 191.

[5] Y. Li , D. Zhang , S. Huang , H. Y. Yang , Nano Energy 2021, 85,105969.

[6] X. Wang , Z. Zhang , B. Xi , W. Chen , Y. Jia , J. Feng , S. Xiong , ACS Nano 2021, 15, 9244. 34081440 10.1021/acsnano.1c01389

[7] L. E. Blanc , D. Kundu , L. F. Nazar , Joule 2020, 4, 771.

[8] P. He , Q. Chen , M. Yan , X. Xu , L. Zhou , L. Mai , C.‐W. Nan , EnergyChem 2019, 1, 100022.

[9] X. Jia , C. Liu , Z. G. Neale , J. Yang , G. Cao , Chem. Rev. 2020, 120, 7795. 32786670 10.1021/acs.chemrev.9b00628

[10] T. Xiong , Y. Zhang , W. S. V. Lee , J. Xue , Adv. Energy Mater. 2020, 10, 2001769.

[11] N. Zhang , X. Chen , M. Yu , Z. Niu , F. Cheng , J. Chen , Chem. Soc. Rev. 2020, 49, 4203. 32478772 10.1039/c9cs00349e

[12] Y. Zhang , L. Tao , C. Xie , D. Wang , Y. Zou , R. Chen , Y. Wang , C. Jia , S. Wang , Adv. Mater. 2020, 32, 1905923. 10.1002/adma.201905923 31930593

[13] J. Wang , J.‐G. Wang , H. Liu , Z. You , Z. Li , F. Kang , B. Wei , Adv. Funct. Mater. 2021, 31, 2007397.

[14] Y. Fu , Q. Wei , G. Zhang , X. Wang , J. Zhang , Y. Hu , D. Wang , L. Zuin , T. Zhou , Y. Wu , S. Sun , Adv. Energy Mater. 2018, 8, 1801445.

[15] Y. Liu , J. Zhi , M. Sedighi , M. Han , Q. Shi , Y. Wu , P. Chen , Adv. Energy Mater. 2020, 10, 2002578.

[16] X. Zhu , Z. Cao , W. Wang , H. Li , J. Dong , S. Gao , D. Xu , L. Li , J. Shen , M. Ye , ACS Nano 2021, 15, 2971. 33492135 10.1021/acsnano.0c09205

[17] Y. Zhang , F. Wan , S. Huang , S. Wang , Z. Niu , J. Chen , Nat. Commun. 2020, 11, 2199. 32366904 10.1038/s41467-020-16039-5 PMC7198488

[18] X. Yang , W. Deng , M. Chen , Y. Wang , C. F. Sun , Adv. Mater. 2020, 32, 2003592. 10.1002/adma.202003592 33015911

[19] X. Wang , Y. Li , S. Wang , F. Zhou , P. Das , C. Sun , S. Zheng , Z.‐S. Wu , Adv. Energy Mater. 2020, 10, 2000081.

[20] S. Deng , Z. Yuan , Z. Tie , C. Wang , L. Song , Z. Niu , Angew. Chem. Int. Ed. 2020, 59, 22002. 10.1002/anie.202010287 32841453

[21] L. Wang , K.‐W. Huang , J. Chen , J. Zheng , Sci. Adv. 2019, 5, eaax4279. 32047853 10.1126/sciadv.aax4279 PMC6984968

[22] W. Deng , Z. Li , Y. Ye , Z. Zhou , Y. Li , M. Zhang , X. Yuan , J. Hu , W. Zhao , Z. Huang , C. Li , H. Chen , J. Zheng , R. Li , Adv. Energy Mater. 2021, 11, 2003639.

[23] L. Ma , S. Chen , C. Long , X. Li , Y. Zhao , Z. Liu , Z. Huang , B. Dong , J. A. Zapien , C. Zhi , Adv. Energy Mater. 2019, 9, 1902446.

[24] T. Sun , Z. J. Li , Y. F. Zhi , Y. J. Huang , H. J. Fan , Q. Zhang , Adv. Funct. Mater. 2021, 31, 2010049.

[25] Y. Gao , G. Li , F. Wang , J. Chu , P. Yu , B. Wang , H. Zhan , Z. Song , Energy Storage Mater. 2021, 40, 31.

[26] J. Kumankuma‐Sarpong , S. Tang , W. Guo , Y. Fu , ACS Appl. Mater. Interfaces 2021, 13, 4084. 33459008 10.1021/acsami.0c21339

[27] L. Dai , Y. Wang , L. Sun , Y. Ding , Y. Yao , L. Yao , N. E. Drewett , W. Zhang , J. Tang , W. Zheng , Adv. Sci. 2021, 8, 2004995. 10.1002/advs.202004995 PMC8224442 34194938

[28] D. Wang , L. Wang , G. Liang , H. Li , Z. Liu , Z. Tang , J. Liang , C. Zhi , ACS Nano 2019, 13, 10643. 31419380 10.1021/acsnano.9b04916

[29] J. Ji , H. Wan , B. Zhang , C. Wang , Y. Gan , Q. Tan , N. Wang , J. Yao , Z. Zheng , P. Liang , J. Zhang , H. Wang , L. Tao , Y. Wang , D. Chao , H. Wang , Adv. Energy Mater. 2021, 11, 2003203.

[30] Y. Zeng , X. F. Lu , S. L. Zhang , D. Luan , S. Li , X. W. Lou , Angew. Chem. Int. Ed. 2021, 60, 22189. 10.1002/anie.202107697 PMC8518934 34313363

[31] Y. Zhang , S. Deng , M. Luo , G. Pan , Y. Zeng , X. Lu , C. Ai , Q. Liu , Q. Xiong , X. Wang , X. Xia , J. Tu , Small 2019, 15, 1905452. 10.1002/smll.201905452 31608588

[32] J. Huang , Z. Wang , M. Hou , X. Dong , Y. Liu , Y. Wang , Y. Xia , Nat. Commun. 2018, 9, 2906. 30046036 10.1038/s41467-018-04949-4 PMC6060179

[33] G. Fang , C. Zhu , M. Chen , J. Zhou , B. Tang , X. Cao , X. Zheng , A. Pan , S. Liang , Adv. Funct. Mater. 2019, 29, 1808375.

[34] T. Xiong , Z. G. Yu , H. Wu , Y. Du , Q. Xie , J. Chen , Y.‐W. Zhang , S. J. Pennycook , W. S. V. Lee , J. Xue , Adv. Energy Mater. 2019, 9, 1803815.

[35] J. Wang , J. G. Wang , X. Qin , Y. Wang , Z. You , H. Liu , M. Shao , ACS Appl. Mater. Interfaces 2020, 12, 34949. 32680423 10.1021/acsami.0c08812

[36] Q. Tan , X. Li , B. Zhang , X. Chen , Y. Tian , H. Wan , L. Zhang , L. Miao , C. Wang , Y. Gan , J. Jiang , Y. Wang , H. Wang , Adv. Energy Mater. 2020, 10, 2001050.

[37] L. Liu , Y.‐C. Wu , L. Huang , K. Liu , B. Duployer , P. Rozier , P.‐L. Taberna , P. Simon , Adv. Energy Mater. 2021, 11, 2101287.

[38] Q. Zhao , A. Song , W. Zhao , R. Qin , S. Ding , X. Chen , Y. Song , L. Yang , H. Lin , S. Li , F. Pan , Angew. Chem. Int. Ed. 2021, 60, 4169. 10.1002/anie.202011588 33124115

[39] Y. Li , M. Chen , B. Liu , Y. Zhang , X. Liang , X. Xia , Adv. Energy Mater. 2020, 10, 2000927.

[40] D. Zhang , J. Cao , X. Zhang , N. Insin , S. Wang , J. Han , Y. Zhao , J. Qin , Y. Huang , Adv. Funct. Mater. 2021, 31, 2009412.

[41] S. Lian , C. Sun , W. Xu , W. Huo , Y. Luo , K. Zhao , G. Yao , W. Xu , Y. Zhang , Z. Li , K. Yu , H. Zhao , H. Cheng , J. Zhang , L. Mai , Nano Energy 2019, 62, 79.

[42] Y. Fang , Y. Zeng , Q. Jin , X. F. Lu , D. Luan , X. Zhang , X. W. Lou , Angew. Chem. Int. Ed. 2021, 60, 8515. 10.1002/anie.202100471 33481323

[43] B. Wu , G. Zhang , M. Yan , T. Xiong , P. He , L. He , X. Xu , L. Mai , Small 2018, 14, 1703850. 10.1002/smll.201703850 29392874

[44] Z. Zhang , S. Li , B. Zhao , X. Zhang , X. Wang , Z. Wen , S. Ji , J. Sun , J. Phys. Chem. C 2021, 125, 20195.

[45] Y. Zhang , Y. Liu , Z. Liu , X. Wu , Y. Wen , H. Chen , X. Ni , G. Liu , J. Huang , S. Peng , J. Energy Chem. 2022, 64, 23.

[46] Y. Jiang , D. Ba , Y. Li , J. Liu , Adv. Sci. 2020, 7, 1902795. 10.1002/advs.201902795 PMC7080538 32195094

[47] C. Chen , M. Shi , Y. Zhao , C. Yang , L. Zhao , C. Yan , Chem. Eng. J. 2021, 422, 130375.

[48] G. Wang , Y. Wang , B. Guan , J. Liu , Y. Zhang , X. Shi , C. Tang , G. Li , Y. Li , X. Wang , L. Li , Small 2021, 17, 2104557. 10.1002/smll.202104557 34643326

[49] R. Liang , J. Fu , Y.‐P. Deng , Y. Pei , M. Zhang , A. Yu , Z. Chen , Energy Storage Mater. 2021, 36, 478.

[50] J. Han , H. Euchner , M. Kuenzel , S. M. Hosseini , A. Groß , A. Varzi , S. Passerini , ACS Energy Lett. 2021, 6, 3063.

[51] Y. Zeng , X. Zhang , Y. Meng , M. Yu , J. Yi , Y. Wu , X. Lu , Y. Tong , Adv. Mater. 2017, 29, 1700274. 10.1002/adma.201700274 28452147

[52] W. Ju , A. Bagger , G. P. Hao , A. S. Varela , I. Sinev , V. Bon , B. Roldan Cuenya , S. Kaskel , J. Rossmeisl , P. Strasser , Nat. Commun. 2017, 8, 944. 29038491 10.1038/s41467-017-01035-z PMC5643516

[53] L. Yang , S. Cheng , J. Wang , X. Ji , Y. Jiang , M. Yao , P. Wu , M. Wang , J. Zhou , M. Liu , Nano Energy 2016, 30, 293.

[54] S. Cheng , L. Yang , D. Chen , X. Ji , Z.‐j. Jiang , D. Ding , M. Liu , Nano Energy 2014, 9, 161.

[55] P. Shang , Y. Liu , Y. Mei , L. Wu , Y. Dong , Mater. Chem. Front. 2021, 5, 8002.

[56] N. Zhang , F. Cheng , J. Liu , L. Wang , X. Long , X. Liu , F. Li , J. Chen , Nat. Commun. 2017, 8, 405. 28864823 10.1038/s41467-017-00467-x PMC5581336

[57] R. Trocoli , F. La Mantia , ChemSusChem 2015, 8, 481. 25510850 10.1002/cssc.201403143

[58] L. Zhang , L. Chen , X. Zhou , Z. Liu , Adv. Energy Mater. 2015, 5, 1400930.

[59] N. Qiu , H. Chen , Z. Yang , S. Sun , Y. Wang , Electrochim. Acta 2018, 272, 154.

[60] D. Kundu , B. D. Adams , V. Duffort , S. H. Vajargah , L. F. Nazar , Nat. Energy 2016, 1, 16119.

[61] B. Lee , C. S. Yoon , H. R. Lee , K. Y. Chung , B. W. Cho , S. H. Oh , Sci. Rep. 2014, 4, 6066. 25317571 10.1038/srep06066 PMC5377529

[62] W. Sun , F. Wang , S. Hou , C. Yang , X. Fan , Z. Ma , T. Gao , F. Han , R. Hu , M. Zhu , C. Wang , J. Am. Chem. Soc. 2017, 139, 9775. 28704997 10.1021/jacs.7b04471

[63] H. Pan , Y. Shao , P. Yan , Y. Cheng , K. S. Han , Z. Nie , C. Wang , J. Yang , X. Li , P. Bhattacharya , K. T. Mueller , J. Liu , Nat. Energy 2016, 1, 16039.

[64] Y. Huang , J. Mou , W. Liu , X. Wang , L. Dong , F. Kang , C. Xu , Nano‐Micro Lett. 2019, 11, 49. 10.1007/s40820-019-0278-9 PMC7770901 34138004

[65] X. Guo , J. Zhou , C. Bai , X. Li , G. Fang , S. Liang , Mater. Today Energy 2020, 16, 100396.

[66] C. Liu , Z. Neale , J. Zheng , X. Jia , J. Huang , M. Yan , M. Tian , M. Wang , J. Yang , G. Cao , Energy Environ. Sci. 2019, 12, 2273.

[67] J. Halim , K. M. Cook , M. Naguib , P. Eklund , Y. Gogotsi , J. Rosen , M. W. Barsoum , Appl. Surf. Sci. 2016, 362, 406.

