# Supplementary material for: Synthesis of Nitrogen‐Doped KMn8O16 with Oxygen Vacancy for Stable Zinc‐Ion Batteries
Source: Adv Sci (Weinh). 2022 Feb 10;9(10):2106067. doi: 10.1002/advs.202106067 (PMC8981436; doi:10.1002/advs.202106067)
Supplement: Supplementary file 1 — Supporting Information [file ADVS-9-2106067-s001.pdf]

ADVANCED SCIENCE
Open Access

Supporting Information

for *Adv. Sci.*, DOI 10.1002/advs.202106067

Synthesis of Nitrogen-Doped KMn_8O_{16} with Oxygen Vacancy for Stable Zinc-Ion Batteries

Guodong Cui, Yinxiang Zeng, Jinfang Wu, Yan Guo, Xiaojun Gu* and Xiong Wen (David) Lou**

ADVANCED SCIENCE
Open Access

Supporting Information

for *Adv. Sci.*, DOI: 10.1002/advs.202106067

Synthesis of Nitrogen-doped KMn_8O_{16} with Oxygen Vacancy for Stable Zinc-Ion Batteries

Guodong Cui, Yinxiang Zeng, Jinfang Wu, Yan Guo, Xiaojun Gu,* and Xiong Wen (David) Lou**

Supporting Information *for*

Synthesis of Nitrogen-doped KMn_8O_{16} with Oxygen Vacancy for Stable Zinc-Ion Batteries

Guodong Cui,[†] *Yinxiang Zeng,*[†] *Jinfang Wu, Yan Guo,* Xiaojun Gu,* and Xiong Wen (David) Lou**

[*] G. D. Cui, J. F. Wu, Prof. Y. Guo, Prof. X. J. Gu

School of Chemistry and Chemical Engineering, Inner Mongolia University, Hohhot 010021, China

E-mail: guoyan@imu.edu.cn (Y. Guo), xiaojun.gu@imu.edu.cn (X. Gu)

Dr. Y. X. Zeng, Prof. X. W. Lou

School of Chemical and Biomedical Engineering, Nanyang Technological University, 62 Nanyang Drive, Singapore, 637459, Singapore

Email: xwlou@ntu.edu.sg; Webpage: https://personal.ntu.edu.sg/xwlou/

[†] These authors contribute equally.

Experimental Section

Synthesis of C_3N_4: In a typical process, the C_3N_4 was synthesized via thermal polymerization of urea.[1] The urea was calcinated in a tube furnace, and heated to 515 ^{o}C for 4 h with a heating rate of 2.5 ^{o}C min^{-1} under Ar flow. After cooling down to room temperature, the light yellow C_3N_4 powder was obtained by grinding the bulk sample.

Synthesis of N-KMO: N-KMO was synthesized using a hydrothermal method. Firstly, 1.5 mmol of $KMnO_4$ was dissolved into 35 mL of deionized water under magnetic stirring until it was completely dissolved. Subsequently, 100 mg of C_3N_4 was added into the above solution and stirred for 30 min. The mixture was then transferred into a 50 mL autoclave, sealed and maintained at 180 ^{o}C for 12 h in an oven. After cooling down to room temperature, the black product (named as N-KMO) was collected by centrifugation and then washed with deionized water and absolute ethanol for several times, followed by drying at 80 ^{o}C for 12 h.

Synthesis of O_v-MnO_{2-x}: α-MnO_2 with oxygen vacancy (named as O_v-MnO_{2-x}) was synthesized according to the reference.[2] Typically, 2.5 mmol of $KMnO_4$ was firstly dissolved into 40 mL of deionized water under magnetic stirring until it was completely dissolved, and then 1.0 mL of concentrated HCl was added into the above solution under vigorous stirring at room temperature. The solution was transferred into a 50 mL Teflon autoclave, sealed and maintained at 160 ^{o}C for 12 h. After cooling to room temperature, the brown product was collected by centrifugation and then washed with deionized water and absolute ethanol for several times, followed by drying at 80 ^{o}C for 12 h.

Synthesis of MnO_2: For a comparison, a MnO_2 sample was prepared by annealing the above obtained O_v-MnO_{2-x} at 200 ^{o}C for 10 h in air atmosphere.

Characterizations: The morphologies were investigated using a high-resolution transmission electron microscopy (HRTEM, Philips Tecnai G2 F20) and a scanning electron microscope (SEM, S-4800). The chemical composition of the samples was characterized by an element analyzer (EA, Elem vario EL cube) and X-ray photoelectron spectroscopy (XPS， ESCALABXI+). Structure characterization was determined by X-ray diffraction (XRD) using a D/max 2500 v/PC system (Rigaku Company) with CuKα radiation (λ=1.5406 Å). Porous structures of the samples were characterized by nitrogen adsorption measurements at 77 K (ASAP 2020, Micromeritics, USA). The electron paramagnetic resonance (EPR) spectra were recorded on a Bruker EMXplus electron

paramagnetic resonance spectrometer at room temperature. Raman analysis was performed on a RENISHAW inVia Microscope Raman under 532 nm excitation. Inductively coupled plasma optical emission spectroscopy (ICP-OES) spectrometer (720ES) was used to determine the concentration of Mn element in a 2 M $ZnSO_4$ aqueous electrolyte. The conductivity was measured by powder resistivity tester (FM100GH).

Electrochemical measurements: The N-KMO cathode was prepared by mixing N-KMO as the active material, acetylene black as the electrical conductor and Polyvinylidene fluoride (PVDF) as the binder at a mass ratio of 7:2:1 in N-methyl-2-pyrrolidone solvent. Then, the obtained slurry was coated on the carbon paper. The mass loading of active material in each electrode disc was around 1 mg cm^{-2}. The aqueous solution (about 0.5 mL in each battery) containing 2 M $ZnSO_4$ and 0.1 M $MnSO_4$ was employed as the electrolyte. Glass fiber membrane (GF/D) and zinc foil (0.2 mm in thickness) were used as the separator and anode, respectively. For comparison, the O_V-MnO_{2-x} and MnO_2 cathodes were prepared using the same method to assemble batteries. All electrochemical characterizations were tested using coin type button batteries (CR2430). The galvanostatic discharge/charge (GCD) was tested by using a battery test system (LAND, CT3001A) under the voltage window of 0.8-1.8 V. Cyclic voltammetric (CV) and electrochemical impedance spectroscopy (EIS) were tested by using an electrochemical workstation (CHI 660E). Calculated the mass specific discharge capacity (mAh g^{-1}) of the battery according to the equation of $C=\frac{I\times\Delta t}{m}$, where I is discharge current (mA), Δt is discharge time (h), and m is the mass (g) of active material on carbon paper. The energy density (Wh kg^{-1}) of battery was calculated by equation of $E=C\times\Delta V$, where ΔV is voltage. The power density (W kg^{-1}) of battery was calculated by equation of $P=\frac{E}{\Delta t}$, where Δt is discharge time (h).[3] The ions diffusion coefficient (cm^2 s^{-1}) was tested by using galvanostatic inermittent titration technique (GITT) and calculated by the following equation:[4]

$$D^{GITT}=\frac{4}{\pi\tau}\left(\frac{m_B V_M}{M_B S}\right)^2\left(\frac{\Delta E_s}{\Delta E_t}\right)^2$$
$$=\frac{4}{\pi\tau}L^2\left(\frac{\Delta E_s}{\Delta E_t}\right)^2$$

where τ (s) is the constant current pulse time, L (cm) is the thickness of the active material coated on the carbon paper, measured by a screw micrometer, ΔE_S (V) is the steady-state voltage change caused by the current pulse, and ΔE_t (V) is voltage changes during the constant current pulse.

Computational methods: Density functional theory (DFT) calculations were carried out in the Cambridge Serial Total Energy Package (CASTEP) program package. The Generalized gradient approximation (GGA) with Perdew-Burke-Ernzerhof (PBE) exchange correlation function was employed to describe the total electronic energy along ultrasoft pseudopotentials. The plane-wave cutoff of 500 eV, Monkhorst-Pack k-point of 4×4×2 and total energy convergence of 2.0×10^{-6} eV were used for the geometry optimization. A 1×1×3 α-MnO_2 supercell containing 28 Mn and 56 O atoms was built. Spin-polarizations are included and all atom coordinates and lattice vectors were fully relaxed in all the calculations. The insertion energy of H^+ into α-MnO_2 were calculated by $E = E_{\partial\text{-}MnO_{2}\text{-}H} - E_{\partial\text{-}MnO_{2}} - E_{H}$, where, $E_{\partial\text{-}MnO_{2}\text{-}H}$, $E_{\partial\text{-}MnO_{2}}$ and E_{H} were total energy for the α-MnO_2 with insertion of H^+, the isolated α-MnO_2 supercell and the isolated H^+, respectively.

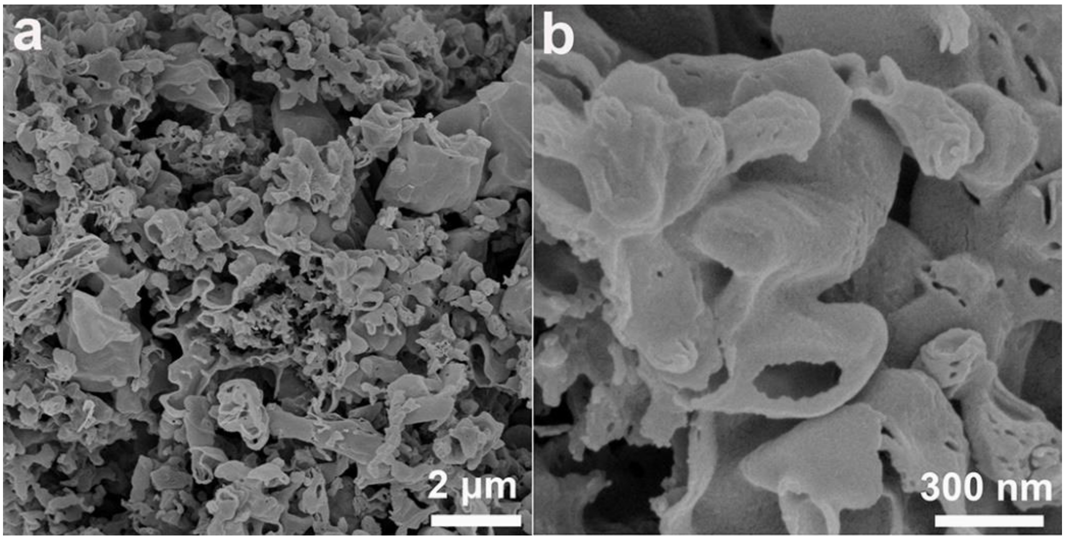

Figure S1. FESEM images of C_3N_4.

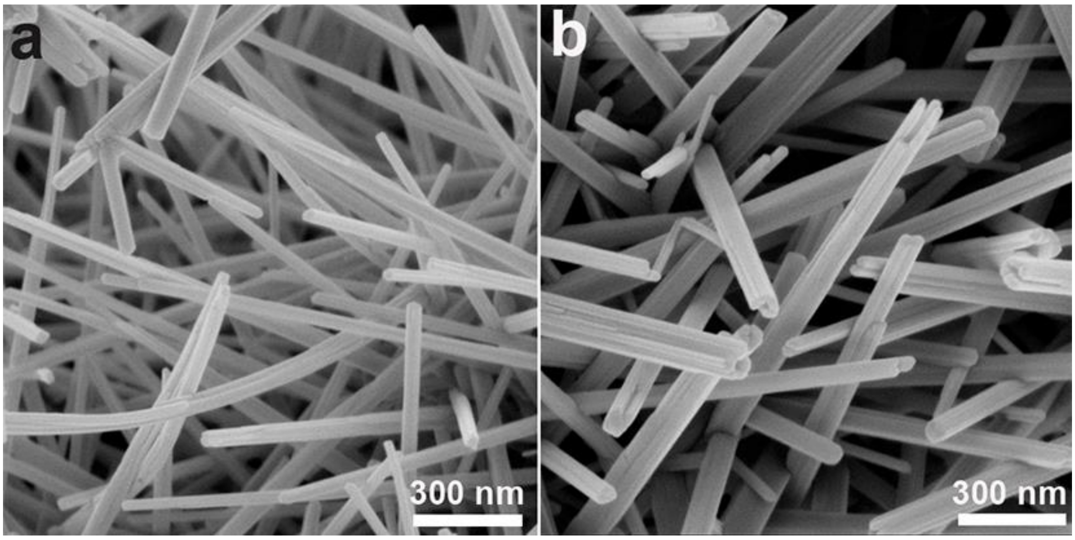

Figure S2. FESEM images of (a) O_v-MnO_{2-x} and (b) MnO_2.

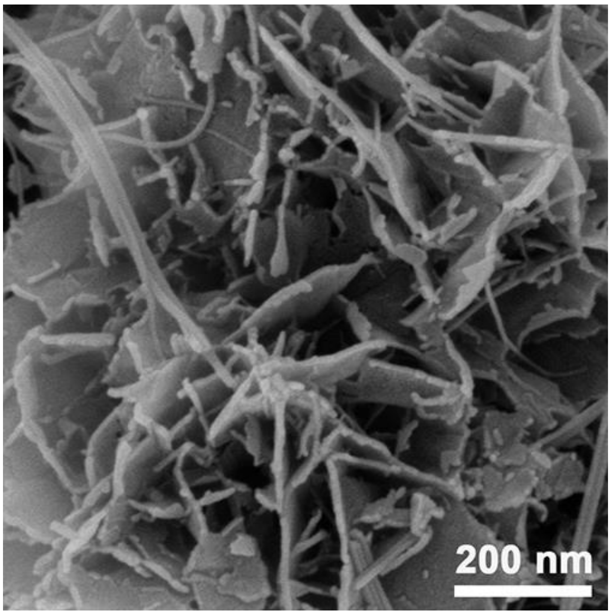

Figure S3. FESEM image of N-KMO.

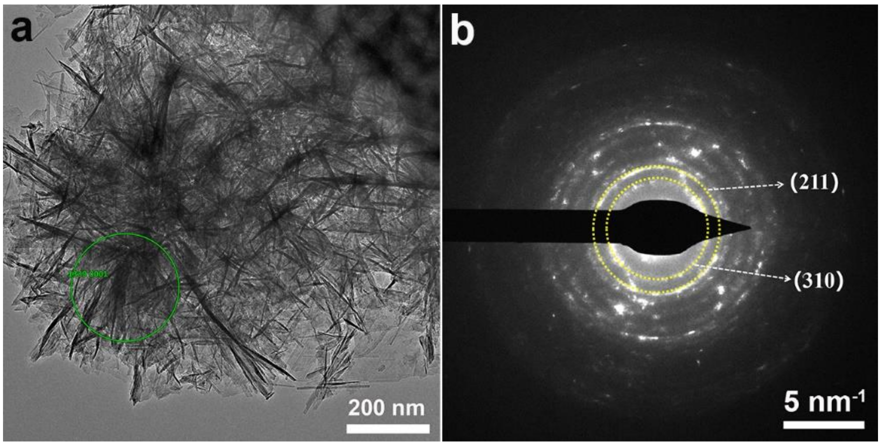

Figure S4. (a) TEM image and (b) the corresponding SAED pattern of N-KMO.

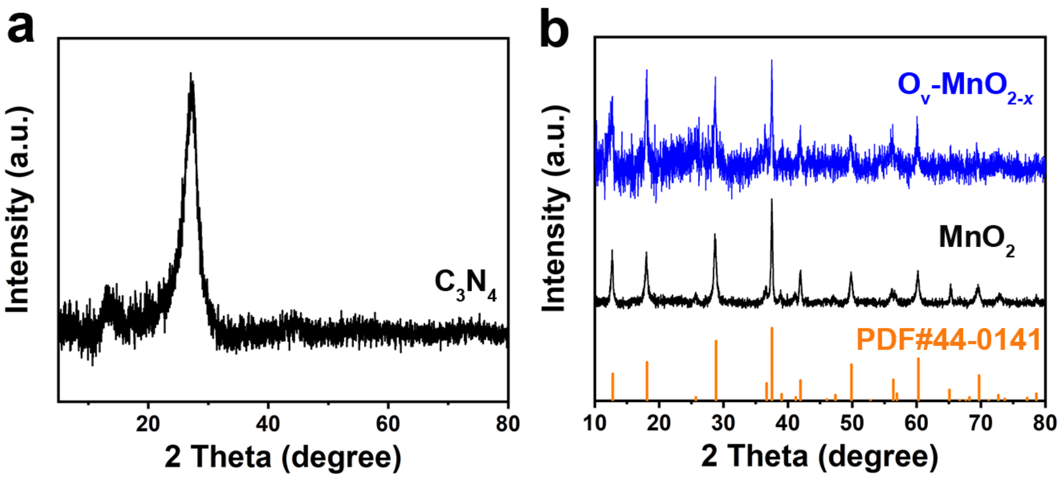

Figure S5. XRD patterns of (a) C_3N_4, (b) O_v-$MnO_{2\text{-}x}$ and MnO_2.

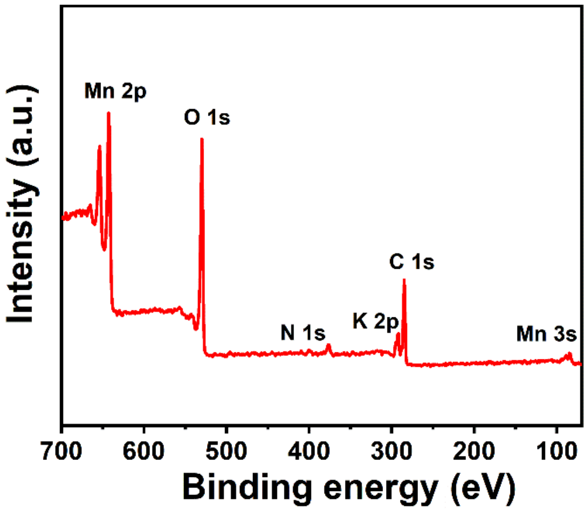

Figure S6. XPS survey spectrum of N-KMO.

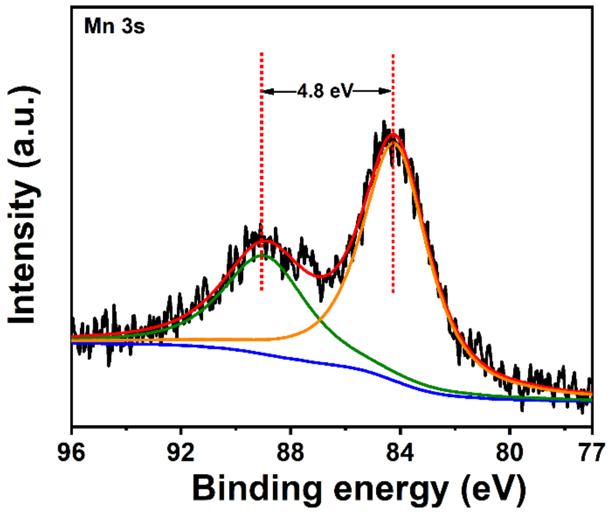

Figure S7. High-resolution XPS spectrum of Mn 3s for N-KMO.

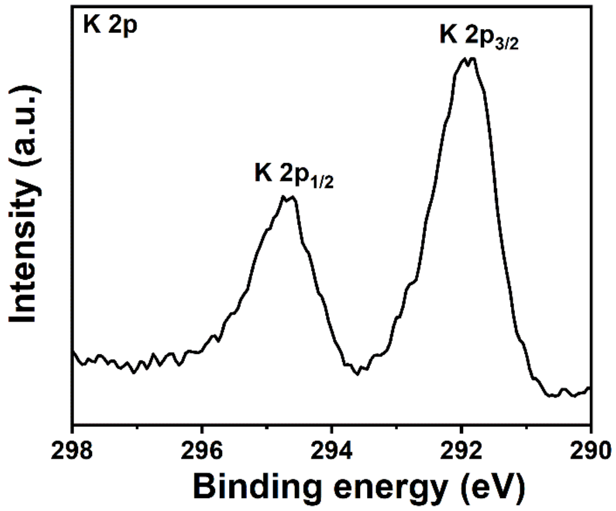

Figure S8. High-resolution XPS spectrum of K 2p for N-KMO.

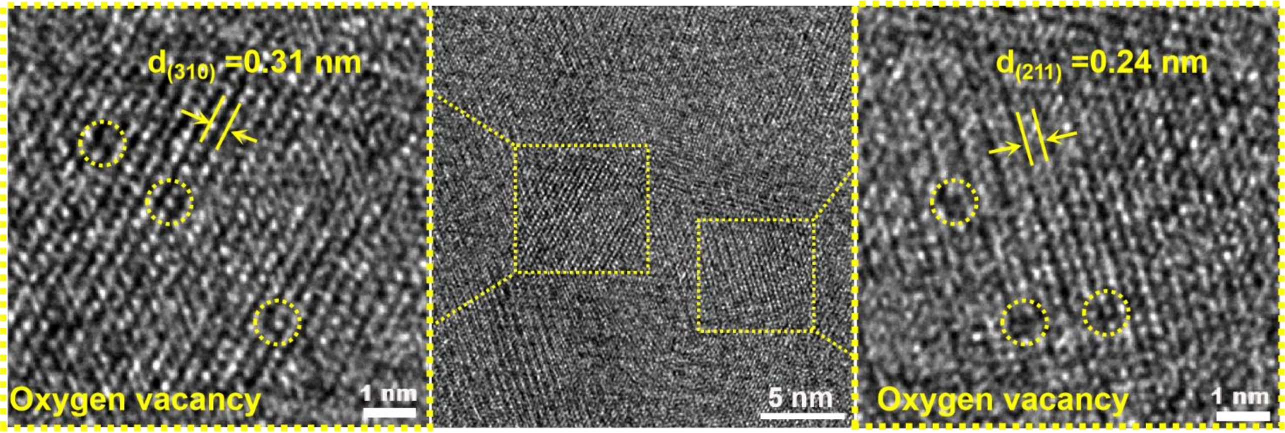

Figure S9. The HRTEM images of N-KMO.

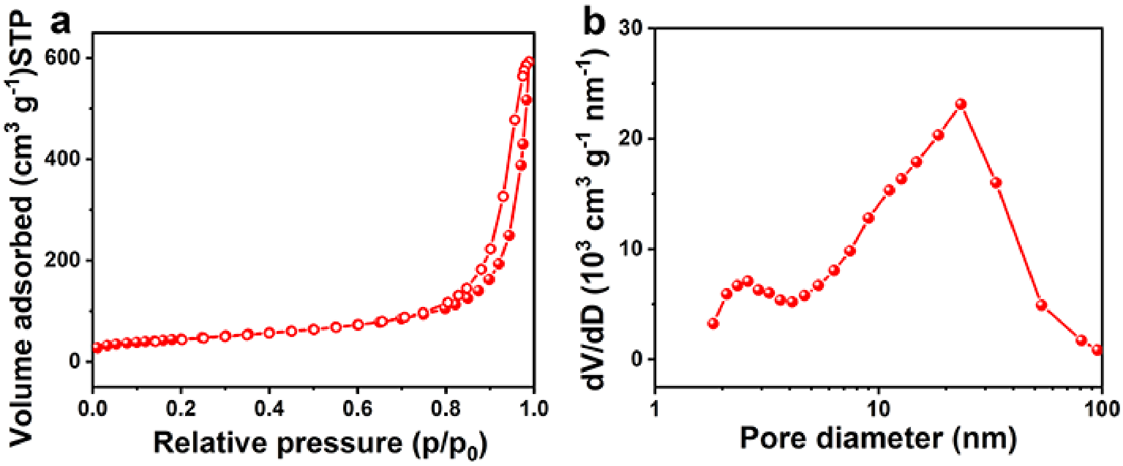

Figure S10. (a) Nitrogen adsorption-desorption isotherm and (b) the pore size distribution curve of N-KMO.

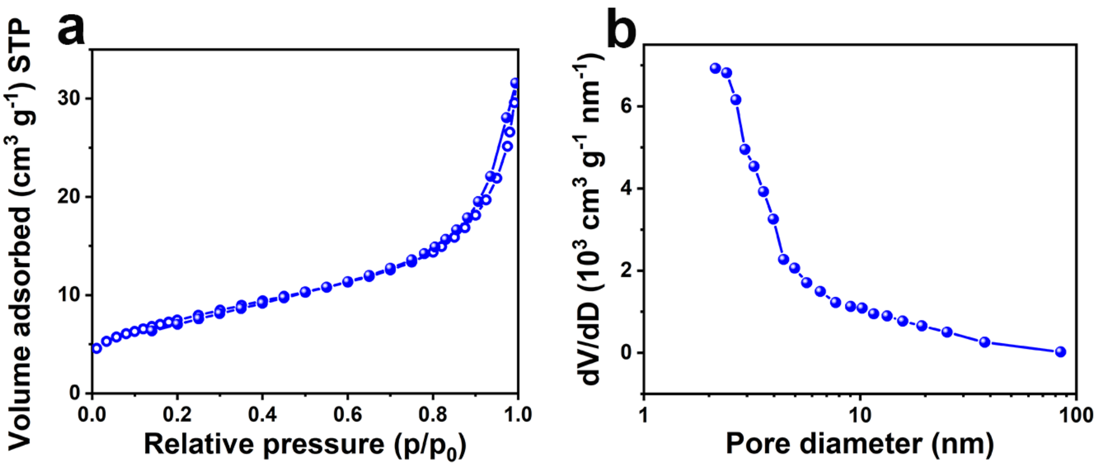

Figure S11. (a) Nitrogen adsorption-desorption isotherm and (b) the pore size distribution curve of O_V-MnO_{2-x}.

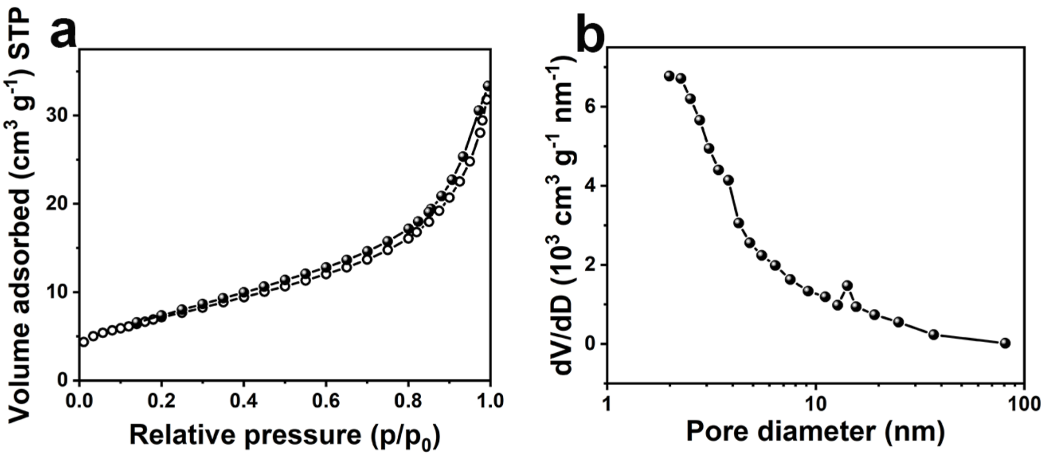

Figure S12. (a) Nitrogen adsorption-desorption isotherms and (b) the pore size distribution curve of MnO_2.

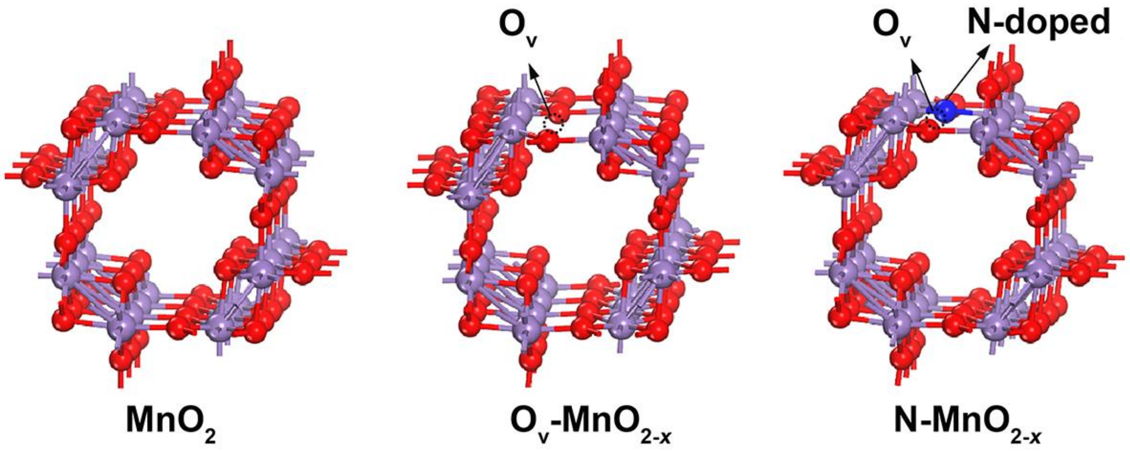

Figure S13. Three geometry optimized configurations: Mn (purple), O (red) and N (blue).

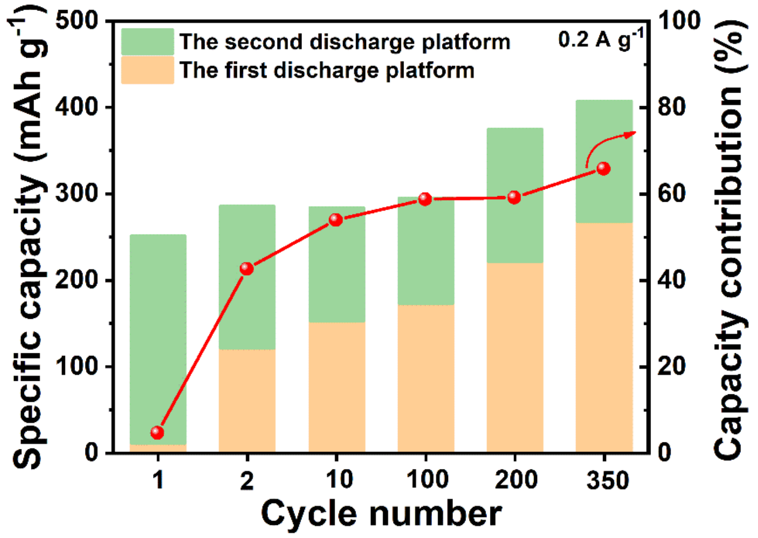

Figure S14. Specific capacity contribution of N-KMO at 0.2 A g^{-1} by the first and second discharge platforms and capacity contribution ratio of the first discharge platform.

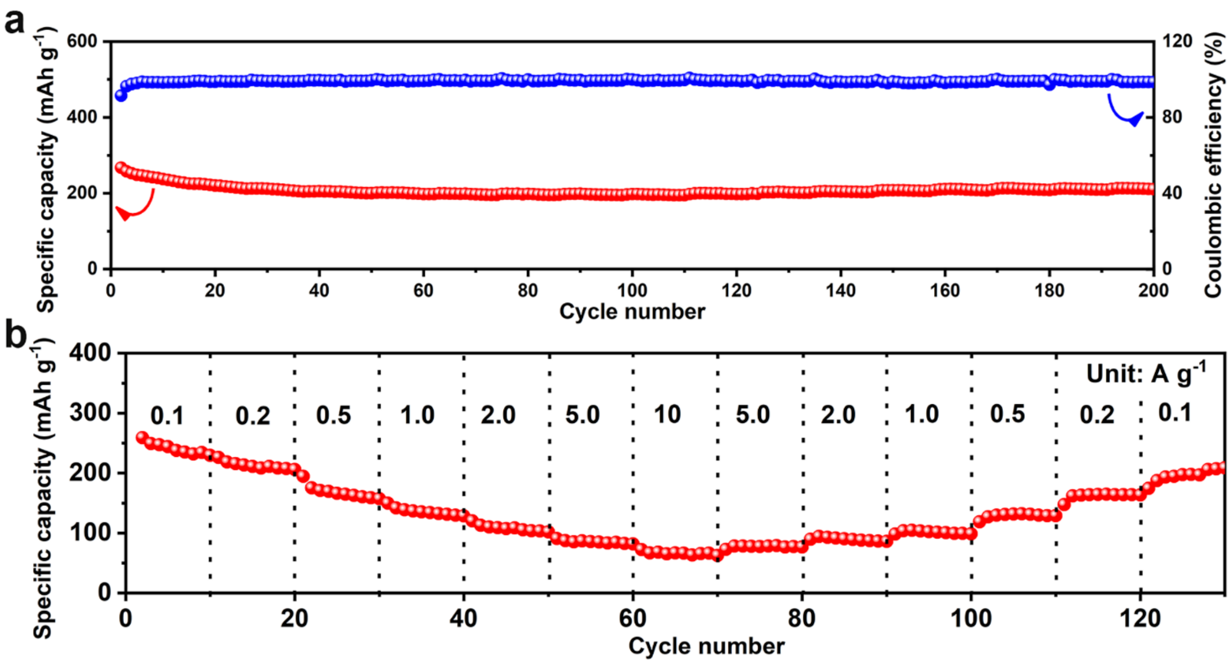

Figure S15. (a) Cyclic performance tested at 0.2 A g^{-1} and (b) rate performance for the N-KMO with active material of around 3.5 mg cm^{-2}.

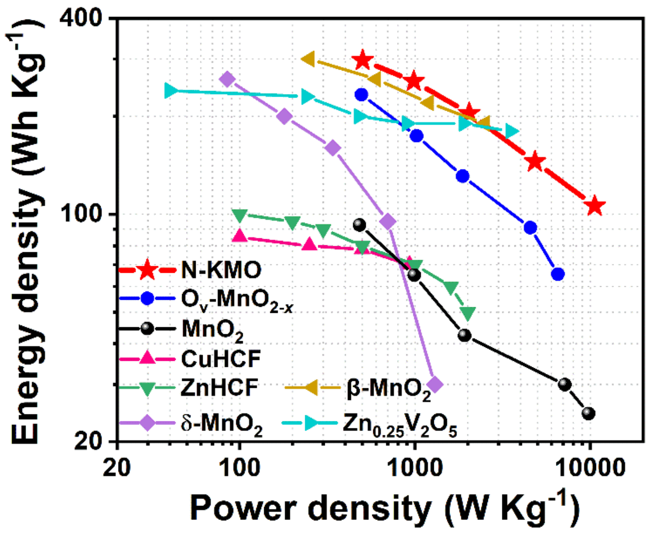

Figure S16. Ragone plots of N-KMO, O_V-MnO_{2-x}, MnO_2 and other reported cathodes used in AZIBs.

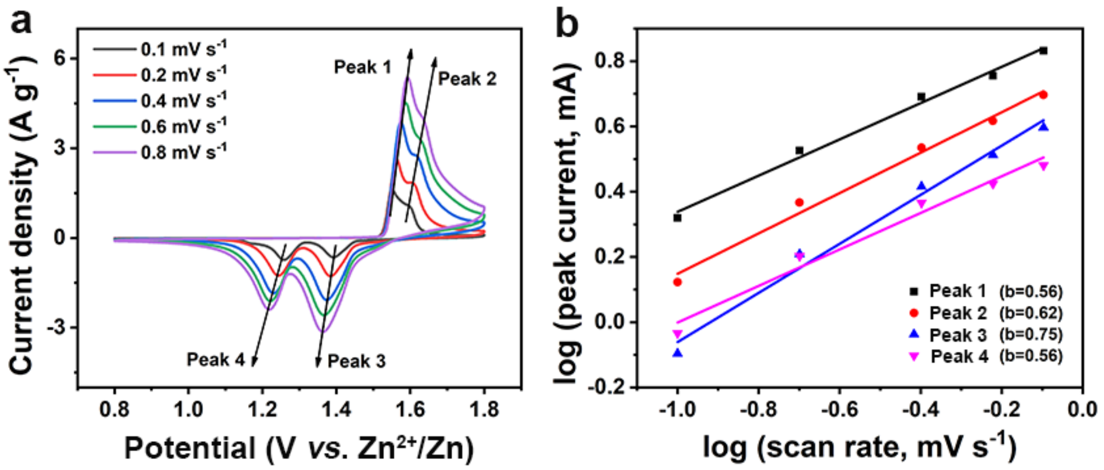

Figure S17. (a) CV curves of O_V-MnO_{2-x} at different scan rates. (b) Determination of the *b* value of O_V-MnO_{2-x} using the relationship between peak current and scan rate.

Table S1. Comparison for electrochemical performances of these representative Mn-based cathode materials used in AZIBs.

Cathode material	Voltage window	Rate performance	Cycling performance	Ref.
N-KMO	0.8-1.8 V	327 mAh g^{-1} at 0.1 A g^{-1}, 106 mAh g^{-1} at 10 A g^{-1}	262 mAh g^{-1} after 2500 cycles at 1 A g^{-1}; 407 mAh g^{-1} after 350 cycles at 0.2 A g^{-1}	This work
α-MnO_2	1.0-1.8 V	285 mAh g^{-1} at 0.103 A g^{-1}, 113 mAh g^{-1} at 3.08 A g^{-1}	92 mAh g^{-1} after 5000 cycles at 1.54 A g^{-1}	5
$ZnMn_2O_4$/C	0.8-1.9V	150 mAh g^{-1} at 0.05 A g^{-1}, 72 mAh g^{-1} at 2 A g^{-1}	80 mAh g^{-1} after 500 cycles at 0.5 A g^{-1}	6
PANI-δ-MnO_2	1.0-1.8V	280 mAh g^{-1} at 0.2 A g^{-1}, 110 mAh g^{-1} at 3 A g^{-1}	280 mAh g^{-1} after 200 cycles at 0.2 A g^{-1}	7
MnO_x@N-C	0.8-1.8V	194 mAh g^{-1} at 0.1 A g^{-1}, 123 mAh g^{-1} at 2 A g^{-1}	195 mAh g^{-1} after 1600 cycles at 1 A g^{-1}	8
Ca_2MnO_4	0.8-1.8V	250 mAh g^{-1} at 0.1 A g^{-1}, 120 mAh g^{-1} at 1 A g^{-1}	100 mAh g^{-1} after 1000 cycles at 1 A g^{-1}	9
$K_{0.8}Mn_8O_{16}$	1.0-1.8V	300 mAh g^{-1} at 0.1 A g^{-1}, 100 mAh g^{-1} at 2 A g^{-1}	150 mAh g^{-1} after 1000 cycles at 1 A g^{-1}	10
MnO_2/Graphene	1.0-1.9V	317 mAh g^{-1} at 0.1 A g^{-1}, 112 mAh g^{-1} at 7.5 A g^{-1}	175 mAh g^{-1} after 600 cycles at 1 A g^{-1}	11
$K_{0.27}MnO_2 \cdot 0.54H_2O$	0.7-1.8V	280 mAh g^{-1} at 0.1 A g^{-1}, 90 mAh g^{-1} at 3 A g^{-1}	84 mAh g^{-1} after 1000 cycles at 3 A g^{-1}	12
d-MnO_2	1.0-1.8V	303 mAh g^{-1} at 0.06 A g^{-1}, 198 mAh g^{-1} at 1.23 A g^{-1}	241 mAh g^{-1} after 250 cycles at 0.308 A g^{-1}	13
MnO_2	1.0-1.85V	275 mAh g^{-1} at 0.3 A g^{-1}, 121 mAh g^{-1} at 3 A g^{-1}	230 mAh g^{-1} after 500 cycles at 1 A g^{-1}	14

References

[1] S. Cao, J. Low, J. Yu, M. Jaroniec, *Adv. Mater.* **2015**, *27*, 2150-2176.

[2] W. M. Chen, L. Qie, Q. G. Shao, L. X. Yuan, W. X. Zhang, Y. H. Huang, *ACS Appl. Mater. Interfaces* **2012**, *4*, 3047-3053.

[3] X. Liang, J. Hao, B. Tan, X. Lu, W. Li, *J. Power Sources* **2020**, *472*, 228507.

[4] Q. Tan, X. Li, B. Zhang, X. Chen, Y. Tian, H. Wan, L. Zhang, L. Miao, C. Wang, Y. Gan, J. Jiang, Y. Wang, H. Wang, *Adv. Energy Mater.* **2020**, *10*, 2001050.

[5] H. Pan, Y. Shao, P. Yan, Y. Cheng, K. S. Han, Z. Nie, C. Wang, J. Yang, X. Li, P. Bhattacharya, K. T. Mueller, J. Liu, *Nat. Energy* **2016**, *1*, 16039.

[6] N. Zhang, F. Cheng, Y. Liu, Q. Zhao, K. Lei, C. Chen, X. Liu, J. Chen, *J. Am. Chem. Soc.* **2016**, *138*, 12894-12901.

[7] J. Huang, Z. Wang, M. Hou, X. Dong, Y. Liu, Y. Wang, Y. Xia, *Nat. Commun.* **2018**, *9*, 2906.

[8] Y. Fu, Q. Wei, G. Zhang, X. Wang, J. Zhang, Y. Hu, D. Wang, L. Zuin, T. Zhou, Y. Wu, S. Sun, *Adv. Energy Mater.* **2018**, *8*, 1801445.

[9] S. Guo, S. Liang, B. Zhang, G. Fang, D. Ma, J. Zhou, *ACS Nano* **2019**, *13*, 13456-13464.

[10] G. Fang, C. Zhu, M. Chen, J. Zhou, B. Tang, X. Cao, X. Zheng, A. Pan, S. Liang, *Adv. Funct. Mater.* **2019**, *29*, 1808375.

[11] J. Wang, J.-G. Wang, H. Liu, Z. You, Z. Li, F. Kang, B. Wei, *Adv. Funct. Mater.* **2021**, *31*, 2007397.

[12] L. Liu, Y. C. Wu, L. Huang, K. Liu, B. Duployer, P. Rozier, P. L. Taberna, P. Simon, *Adv. Energy Mater.* **2021**, *11*, 2101287.

[13] P. Shang, Y. Liu, Y. Mei, L. Wu, Y. Dong, *Mater. Chem. Front.* **2021**, *5*, 8002-8009.

[14] J. Wang, J.-G. Wang, H. Liu, C. Wei, F. Kang, *J. Mater. Chem. A* **2019**, *7*, 13727-13735.
